# Novel Approach for the Detection of the Vestiges of Testicular mRNA Splicing Errors in Mature Spermatozoa of Japanese Black Bulls

**DOI:** 10.1371/journal.pone.0057296

**Published:** 2013-02-26

**Authors:** Taichi Noda, Mitsuhiro Sakase, Moriyuki Fukushima, Hiroshi Harayama

**Affiliations:** 1 Laboratory of Reproductive Biology, Graduate School of Agricultural Science, Kobe University, Kobe, Japan; 2 Northern Center of Agricultural Technology, General Technological Center of Hyogo Prefecture for Agriculture, Forest and Fishery, Asago, Japan; University Hospital of Münster, Germany

## Abstract

There is a serious problem with the reduction of male reproductive performance of the livestock in the world. We have a hypothesis that the splicing error-caused derivation of aberrant sperm motility-related proteins may be one of its causal factors. It is thought that fresh testicular tissues are necessary for the detection of splicing errors of the mRNA. However, it is difficult to obtain testicular tissues from a number of agriculturally important bulls by surgical methods, because such procedures may have deleterious effects on bulls’ reproductive performance. The aim of this study was to examine the usefulness of mRNA fragments collected from ejaculated spermatozoa as alternative analytical samples for detection of the splicing errors. In the first experiment, we characterized the alternative splicing and splicing error of bull testicular *ADCY10* mRNA which coded the synthase of the regulatory molecule for sperm motility “cAMP”. In testes, the exon 11-lacking variant coding the truncated ADCY10 was derived by alternative splicing. However, splicing errors, which accompanied the frame shift in the second cyclase domain, were occasionally observed in the exon 11-lacking variant. This aberrant variant retained intronic nucleotides (4 bases, CCAG) connecting the initial part of exon 10 due to splicing errors and consequently yielded the cleavage site for a restriction enzyme (*Cac8I*) which recognized the nucleotide sequences (GCNNGC). In the second experiment, we recovered residual testicular mRNA fragments from ejaculated spermatozoa and observed the splicing error-caused derivation of the aberrant variant of *ADCY 10*. Ejaculated spermatozoa conserved mRNA fragments of the exon 11-lacking variant coding exons 9, 10, 12 and 13. Moreover, the above-mentioned aberrant variant of *ADCY10* mRNA fragment was detectable by *Cac8I* digestion treatment using the sperm mRNAs. These results indicate the utility of sperm mRNA fragments for the detection of splicing errors in bull testicular mRNAs.

## Introduction

RNA splicing is carried out by the spliceosome, which is composed of seven types (*e.g.*, U1, U2) of small nuclear ribonucleoprotein particles (snRNPs) and a large number of auxiliary proteins [*e.g.*, U2 auxiliary factor (U2AF), splicing factor (SF) 1] [1]. Three types of intronic sequences (splicing signals) are important in the recognition of the borderline between the exon and the intron. Specifically, U1 snRNP, SF1 (or U2 snRNP) and U2AF recognize the 5′ terminal GU of the intron, nucleotides at the branch point, and the intronic sequences of the 3′ terminal (namely the polypyrimidine tract and the 3′ terminal AG of the intron), respectively [1].

Splicing errors occur occasionally due to incorrect selection of the cryptic splicing signals, which are similar to the true splicing signals [2]. Such splicing errors are also involved in splicing flexibility [2]. Indeed, the cryptic splicing signals are used for removal of the intronic sequences when the true splicing signals are disintegrated, and then the new splicing pattern appears. The splicing flexibility is a critical factor in the derivation of various transcripts from a single gene in the eukaryote, and it plays important roles in genetic evolution and biological variation [2].

Several studies [3]–[5] showed that the derivation of aberrant transcripts is one of the causal factors in the decrease of male reproductive performance in mammals. For example, in the alkylglycerone-phosphate synthase (*Agps*) gene of *blind sterile 2* (*bs2*) mice, the key nucleotide (G) for the true splicing signal, which is located in the +5 position of intron 14, is replaced by another nucleotide (A) [3]. This mutation increases the frequency of the skipping of exons 13–14 or exon 14 in the transcription process, and the resultant aberrant transcripts with the premature stop codon are predominantly expressed in testes. Consequently, the accumulation of *Agps*, which is abnormal in the flavin adenine dinucleotide binding domain, causes a disturbance of testicular function leading to male infertility in the *bs2* mice.

Reduced conception rates in the artificial insemination (AI) programs of cattle, using the frozen-thawed ejaculated spermatozoa, have been observed all over the world [6]–[8] and may be related to various factors including heat stress [9] and the deterioration of sperm tolerance to frozen storage [10]. However, the main causal factors for the reduction of male reproductive performance and sperm quality are unknown. Sperm motility-related proteins include components of the cAMP signaling pathway [such as adenylyl cyclase 10 (ADCY10, also known as “sAC” or “SACY”)] which are synthesized in mammalian testes [11]–[13]. We have a hypothesis that the splicing error-caused derivation of aberrant sperm motility-related proteins is linked to the reduction of male reproductive performance in cattle, and we are focusing our investigation on the components of cAMP signaling cascades. For the exact detection of occurrences of splicing errors, tissues containing fresh mRNAs are absolutely necessary. However, because the agriculturally important bulls (sires) whose reproductive performance is well characterized are usually enrolled in an AI program, there are only rare opportunities to collect fresh testicular tissues from them by the necessary invasive surgical procedures, including biopsies, because such procedures may have deleterious effects on the bulls’ testicular function and reduce the sperm quality. In fact, it is not until the bulls’ retirement from the AI programs that fresh testicular tissues can be collected from most sires. Moreover, such tissues are unsuitable for the accomplishment of the objective in this study, as testicular function may be reduced in the retired bulls. For these reasons, we need alternatives to the fresh testicular tissues as the sample source for the collection of testicular mRNAs. We therefore turned our attention to ejaculated spermatozoa, which can be collected repetitively from all sires by a non-surgical method. Namely, we heeded that mature spermatozoa are unique cells without the gene transcriptional activity [14] and expected the availability of sperm mRNAs which were the residuals of transcripts synthesized in testes [14], [15].

The aim of the present study was to examine the usefulness of residuals of testicular mRNAs coding the sperm motility-related proteins collected from ejaculated spermatozoa for detection of the occurrence of splicing errors. In the first part of this study, we characterized the *ADCY10* variant mRNAs collected from fresh testicular tissues in order to reveal the site where the serious splicing errors occur. In the second part, we attempted to detect the vestiges of the splicing errors in the residuals of testicular mRNAs collected from ejaculated spermatozoa to examine their usefulness as analytical samples.

## Materials and Methods

### Samples

All chemicals were purchased from Wako Pure Chemical Industries (Osaka, Japan), unless otherwise specified. Testes, cauda epididymides, kidneys and livers were obtained from Japanese Black bulls (>1 year old) immediately after slaughter at the local meat processing plant (Kakogawa Meat Center, Kakogawa, Hyogo, Japan). We obtained the permission from this meat processing plant to use these animal parts. This meat processing plant is administered by Kakogawa City Meat Public Corporation in consideration for the animal welfare as well as in compliance with the law. Immediately after the recovery, tissue pieces of testes, kidneys and livers (from five bulls) were rapidly frozen in liquid nitrogen and then stored at –80°C until used. Commercial frozen spermatozoa and freshly ejaculated spermatozoa [from Japanese Black bulls (>1 year old)] were supplied from the Northern Center of Agricultural Technology, General Technological Center of Hyogo Prefecture for Agriculture, Forest and Fishery (Hyogo, Japan) as kind gifts. The frozen spermatozoa (from six bulls) were prepared in the Center as described previously [10]. In brief, ejaculates were diluted immediately after collection with an egg yolk-Tris-citrate extender prewarmed at 35°C. The diluted ejaculates were transferred to the water bucket (25°C) and cooled slowly to 4°C in the refrigerator overnight. In the next morning, they were diluted with an equal volume of the extender containing 14% (v/v) glycerol (cryoprotectant) and then rapidly frozen in the 0.5-mL straws with cold gas from the liquid nitrogen. The cauda epididymidis (from one bull) was transported to the laboratory within 240 min after recovery and then subjected to reflux with phosphate-buffered saline (PBS; 136.9 mM NaCl, 2.7 mM KCl, 8.1 mM Na_2_HPO_4_, 1.5 mM KH_2_PO_4_) containing 0.1% polyvinyl alcohol (PVA, Sigma-Aldrich Co., St. Louis, MO) (PBS-PVA) to obtain freshly epididymal spermatozoa. Freshly ejaculated spermatozoa (from three bulls) were transported at approx. 25°C and then used for the examination within 240 min after collection.

### Extraction of Total RNAs and Synthesis of cDNA

Total RNAs were extracted from the tissue pieces by homogenization using ISOGEN (Nippon Gene Co., Tokyo). The spermatozoa were washed once in a two-step gradient of 2 mL of 60% and 5 mL of 30% isotonic Percoll (GE Healthcare UK, Buckinghamshire, England, UK) that was prepared with PBS, washed once in PBS-PVA by centrifugation. All of the washed sperm samples were observed on a heated stage at 38.5°C under a bright-field microscope with a CCD camera, in order to confirm no contamination with somatic cells (Movie S1). After the confirmation, each of the sperm samples was resuspended in 1 mL of ISOGEN, stirred by a vortex mixer and incubated at 60°C for 20 min.

The obtained total RNAs were reverse-transcribed to cDNAs as described previously [16]. The concentrations of the tissular cDNAs were measured and then their final concentrations were adjusted to 400 µg/mL. The concentrations of the sperm cDNAs were 400.0–846.7 µg/mL (final concentration).

### Reverse Transcription-polymerase Chain Reaction (RT-PCR), Sequential Analyses, Construction of the Recombinants, Colony PCR and Treatment Using a Restriction Enzyme (Cac8I)

The cDNA sequence of the bull *ADCY10* was estimated by a discontiguous megaBLAST against the genome and HTGS databases (accession numbers: NW_003103854, AC163525 and XM_003581912) of *Bos taurus* and human *ADCY10* cDNA sequence (NM_018417) as the query. The putative bull *ADCY10* cDNA sequence was used to design primer sets #1–#10 for RT-PCR (Table S1). The PCR products amplified with primer sets #1–#3 and #5–#10 were purified from gels with a QIAquick Gel Extraction kit (Qiagen, Hilden, Germany) and subjected to nucleotide sequencing (Hokkaido System Science, Sapporo, Japan; http://www.hssnet.co.jp/index_e.htm).

The DNA fragments amplified by RT-PCR using primer set #4 were inserted into the cloning site (*EcoRI*/*HindIII*) of a *pSPT19* vector as described previously [16]. After transformation, to confirm the successful ligation, the recombinants were randomly detached from the LB plate [2% LB broth (Invitrogen Corp., Carlsbad, CA), 0.5% NaCl, 1.5% Bacto-agar (Becton Dickinson, Franklin Lakes, NJ), and 10 ng/mL ampicillin sodium (Nacalai Tesque, Kyoto, Japan)]. They were subsequently used for colony PCR with a forward primer (5′-ACCTTATGTATCATACACAT-3′) for a *pSPT19* vector and a reverse primer of primer set #4 for *ADCY10*. The molecular size of the obtained products by colony PCR is larger by approximately 50 bps than that of the inserted DNAs, because the colony PCR products include the nucleotides coded by plasmids. The inserted parts of recombinant DNAs, including the *ADCY10* variants, were amplified by PCR using primer set #4. The amplified products were purified from the gels and then subjected to sequence analyses (Hokkaido System Science) [16] or digestion treatment by *Cac8I* (New England BioLabs Inc., Ipswich, MA) which recognizes the specific nucleotide sequences (GCN/NGC, http://www.neb.com/nebecomm/products/productr0579.asp) at 38.5°C overnight.

### Northern Blotting

The cRNA probes were obtained by transcription from a recombinant including the product amplified with primer set #11 (Table S1) using a digoxigenin (DIG) RNA Labeling Kit (Roche, Mannheim, Germany). Northern blotting was done as described previously [17].

### Evaluation of the Sperm Motility

The motility of frozen-thawed spermatozoa (from bulls #1–#6), freshly epididymal spermatozoa (from bull #7) and freshly ejaculated spermatozoa (from bull #8) before washing was observed and recorded using a CCD camera (Olympus Corporation, Tokyo, Japan) and a DVD recorder (Toshiba, Tokyo, Japan) on a heated stage at 38.5°C under a bright-field microscope (Movies S2– S3). In addition, motility of frozen-stored spermatozoa (from bulls #1, #3 and #5) immediately after thawing was analyzed using Sperm Motility Analysis System (SMAS, DITECT Corporation, Tokyo, Japan). Specifically, the sperm suspension was adjusted the concentration to 1×10^7^ cells/mL with PBS-PVA. Five-µL aliquot of this suspension was put on a 20-µm chamber slide (Standard Count 2 Chamber Slide, Leja, North Holland, Netherlands) and then sperm motility was recorded on a heated stage at 38.5°C under a phase contrast microscope. Approximately 5 fields per preparation were randomly selected, and then sperm motility was evaluated by *in silico* analyses using a developing parameter for livestock. The analyses were replicated twice for each bull.

### Statistical Analyses

The correlation between the percentages of the bull *ADCY10* variant II mRNA in the frozen-thawed spermatozoa and those of motile spermatozoa (spermatozoa exhibiting flagellar beating) or those of progressively motile spermatozoa immediately after thawing was examined by the simple regression analyses (Excel software with the add-in software Statcel3; OMS, Saitama, Japan).

### Antibody Production and Immunodetection of ADCY10 Variant Ortholog Proteins in Ejaculated Spermatozoa

The production of mouse antiserum to the bull ADCY10 variant ortholog protein was performed as described previously [16]. Briefly, the cDNA fragments of *ADCY10* ortholog variants which were amplified by RT-PCR with primer set #12 were cloned into the host bacteria XL-10, including *pGEX-KG*-expressed vectors. Primer sets #12 was designed to amplify the nucleotides coding the first cyclase domain of bull ADCY10. Next, the expression of the GST fusion ADCY10 ortholog proteins was induced by IPTG, and then the purified recombinant proteins used as the antigenic peptides for the antibody production.

Freshly ejaculated spermatozoa (from two bulls) were washed three times with PBS-PVA and subsequently used for the indirect immunofluorescence as described previously [16]. In brief, the sperm preparations were subjected to the mouse antiserum (1∶30), followed by the FITC-conjugated rabbit anti-mouse immunoglobulins antibody (1∶50, DakoCytomation Denmark A/S, Copenhagen, Denmark). They were observed under a differential interference microscope equipped with epifluorescence [U-MWIB2 mirror unit composed of BP 460–490 excitation filter, DM505 dichroic mirror and BA510IF, emission filter, respectively (Olympus Corporation)].

## Results and Discussion

**Figure 1:**
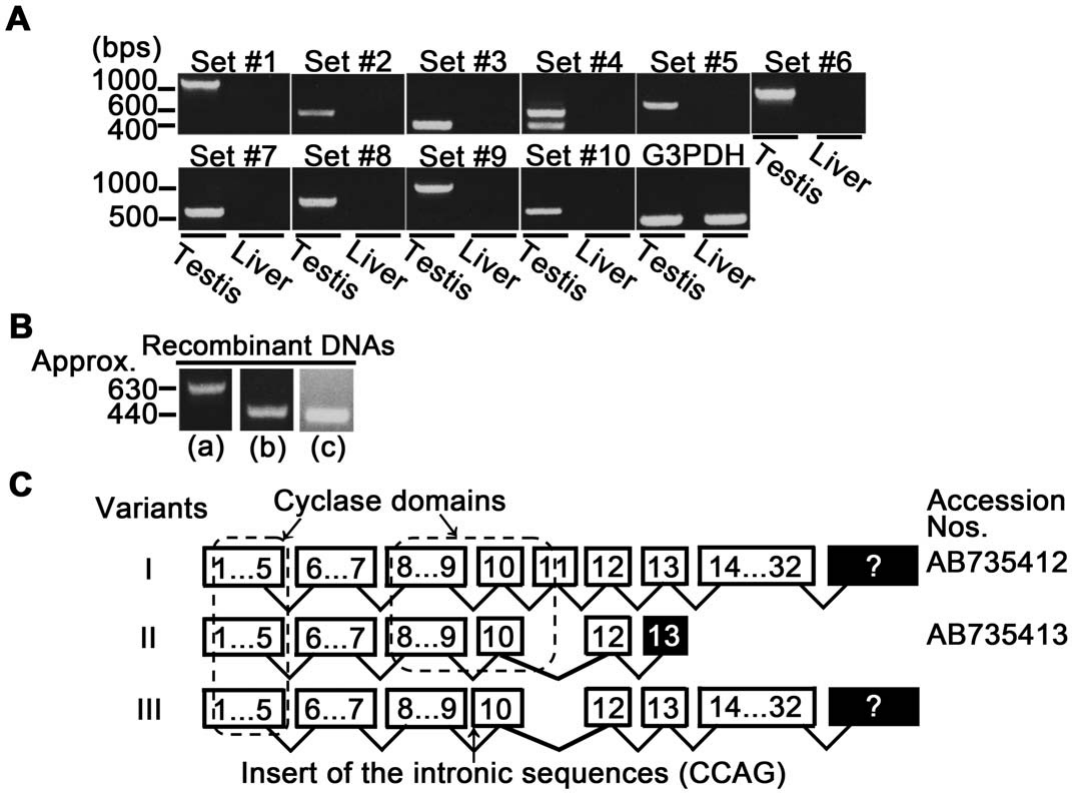
Detection of *ADCY10* variant mRNAs in testes and determination of the site of splicing errors. A: The products amplified by RT-PCR, using ten sets of primers and tissular RNAs, were separated in 1% agarose gels containing 0.01% ethidium bromide. *Glycerol-3-phosphate dehydrogenase* (*G3PDH*) was used as the control. (The panel is representative of three replicates). **B:** Three recombinants (a)–(c) into which the DNA fragments amplified by RT-PCR using primer set #4 were inserted were subjected to the colony PCR with a forward primer for *pSPT19* and a reverse primer of primer set #4. **C:** The numbers surrounded by boxes indicate the numbers of exons of bull *ADCY10*. The variants I, II and III contain the nucleotide sequences of inserted parts of recombinants (a), (b) and (c), respectively. The black-colored boxes show the exons containing the stop codons. The question marks indicate that the exon containing the stop codon remains to be revealed. The parts surrounded by the broken line indicate the exons coding the cyclase domains.

**Figure 2:**
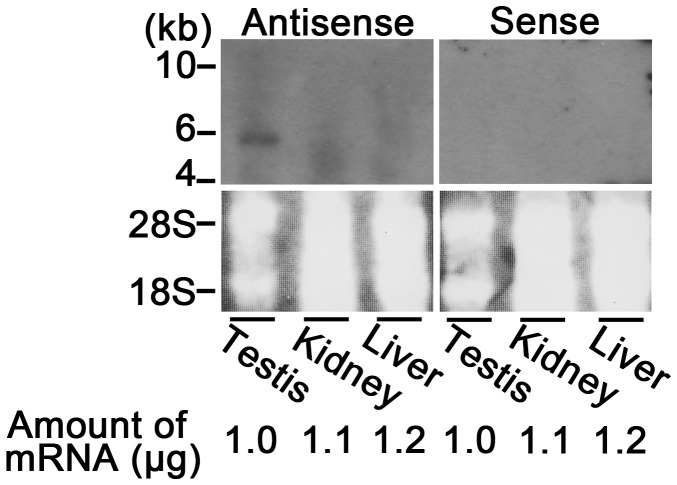
Detection of *ADCY10* variant mRNAs in bull testes by Northern blotting. Total RNAs from testes (1.0 µg), kidneys (1.1 µg) and livers (1.2 µg) were separated by electrophoresis in gels containing formaldehyde and subsequently transferred onto nylon membranes. The obtained membranes were hybridized with either an antisense or sense DIG-labeled cRNA probe which was synthesized with the recombinant DNA into which the product amplified by RT-PCR using primer set #11 was inserted. In addition, the bands of the ribosomal RNAs on the membranes were visualized with a transilluminator. (The panel is representative of three replicates).

Figure 1A shows the results of the detection of bull *ADCY10* variant mRNAs in testes and livers by RT-PCR with 10 primer sets (Table S1). All PCR products were amplified more efficiently in testes than in livers. These results were also supported by Northern blotting using a cRNA probe with affinity to the nucleotide sequences coding the first cyclase domain (Figure 2). These are in agreement with previous observations on rodent [18], [19] and boar testis [20]. In our preliminary experiments, indirect immunofluorescence indicated the presence of ADCY10 variant ortholog proteins in at least spermatids of bull testes (Noda *et al*., unpublished data). It has widely been accepted in mammals that mRNA [19], [21] and protein [11], [22] of this variant show the highest expression in the testis [23]–[26]. In concrete terms, the transcripts and proteins of rodent ADCY10 variants were first detected in the pachytene spermatocytes and accumulated in the germ cells up to the later stages of spermiogenesis [19], [21], [22]. These results suggest that ADCY10 has the potential to take part in the synthesis of cAMP in the testicular germ cells of livestock as well as rodent. In mammals including mice [27], boars [16] and bulls [Noda *et al*., unpublished data], furthermore, the activator type of cAMP-responsive element modulator (CREM) is specifically present in the spermatids and pivotally controls the transcription of spermiogenesis-related proteins (*e.g.*, protamines and transition proteins) [27]–[30]. So, it is possible that cAMP synthesized by ADCY10 may be involved in the gene transcriptional activity in spermatids.

The PCR product amplified with each of primer sets #1–#3 and #5–#10 was detected as a single band and could be used directly for the nucleotide sequencing. However, RT-PCR with primer set #4 amplified at least two products of DNA fragments (Figure 1A). Thus, we decided to construct recombinants into which these PCR products could be inserted for the purpose of their purification.

As shown in Figure 1B, three types of single-band product with the molecular size of lanes (a) approximately 630 bps, (b) approximately 440 bps and (c) approximately 440 bps were amplified by colony PCR using a forward primer for a vector and a reverse primer of primer set #4. The inserted DNAs of recombinants [(a)–(c)] were used for the nucleotide sequencing. Each of the obtained nucleotide sequences of the inserted parts of recombinants [(a)–(c)] was combined with the nucleotide sequences of the products amplified with primer sets #1–3 and #5–10.

These nucleotide sequences were subjected to a megaBLAST against the genome database of *Bos taurus* (BLASTN, http://blast.ncbi.nlm.nih.gov/Blast.cgi). In addition, these nucleotide sequences were converted into the amino acid sequences and then used for discontiguous megaBLAST analyses against the RefSeq protein databases of *Homo sapiens* and *Rattus norvegicus* (BLASTX) and analyses of the conserved domains with CD-search (http://www.ncbi.nlm.nih.gov/Structure/cdd/wrpsb.cgi).

**Figure 3:**
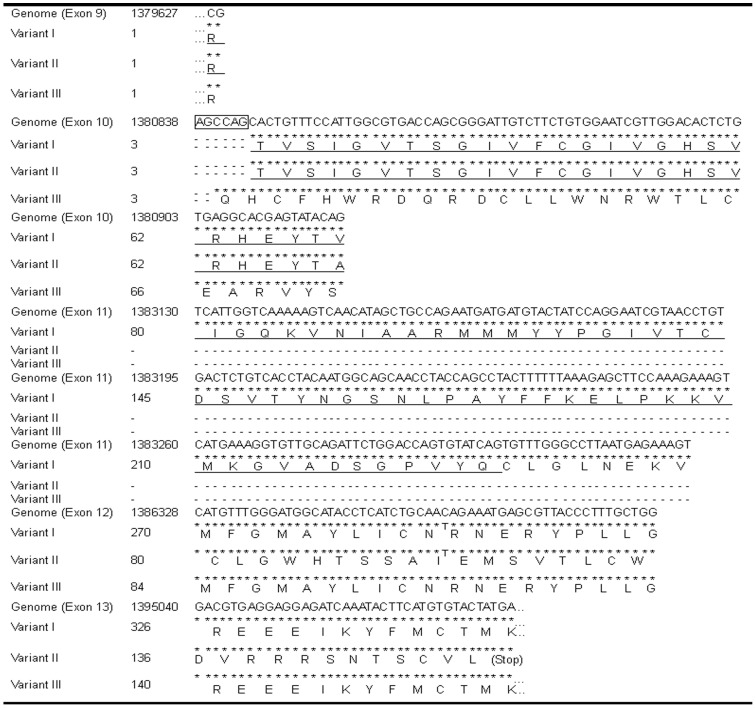
Multiple alignment of nucleotide sequences and amino acid sequences coded by *ADCY10* variants I – III. A part of sequences of nucleotides and amino acids coded by each variant are shown in the upper lanes and lower lanes, respectively. The asterisks mark identical nucleotides between the bull genome sequence (NW_001494712), and bull *ADCY10* variants I, II or III, and the hyphens are the lacking parts of the corresponding nucleotides between these sequences. The nucleotides surrounded by a box show the intronic sequences. “Stop” indicates the nucleotide triplet coding the stop codon. The underlined amino acids indicate the second cyclase domain.

These analyses predicted that the bull *ADCY10* gene is composed of at least 32 exons and that three variants (I–III) of bull *ADCY10* mRNAs are expressed in testes (Figure 1C). Specifically, the sequential homology rates of the protein encoded by the bull variant I were 80% and 81% against the human full-length form of ADCY10 (ADCY10_fl_) protein (accession number: NP_060887) and rat ADCY10_fl_ protein (NP_067716), respectively (Figure S1). Additionally, two cyclase domains of the N-terminal region, which were the landmarks of ADCY10 [18], [31], [32], were conserved in bull variant I (Figure S1). These results indicate the variant I is the ortholog of *ADCY10_fl_*. Variant II was a form lacking exon 11 which corresponded to exon 12 (coding the C-terminal part of the second cyclase domain) of human *ADCY10* (NM_018417) (Figure 3). In this variant, one of the nucleotide triplets was changed to the stop codon in exon 13 by a frame shift which was due to the alternative splicing (Figure 3). Consequently, variant II coded the normal truncated form of ADCY10 (ADCY10_tf_) with two cyclase domains. Variant III was a form retaining the intronic nucleotides (4 bases, CCAG) connecting to the initial part of exon 10 by the splicing error and lacking exon 11 by the alternative splicing (Figure 3). In this variant, the frame shift occurred twice by the splicing error and alternative splicing. Thus, this variant coded the aberrant ADCY10_fl_ protein with abnormal nucleotide triplets in exon 10 which disintegrate the second cyclase domain (Figure 3).

Jaiswal and Conti reported that the *Adcy10* mRNA coding the ADCY10_tf_ is generated in rat and mouse testes by alternative splicing, which results in the lack of exon 11 after the second cyclase domain [corresponding to exon 13 of the human *ADCY10* (NM_018417) and exon 12 of the bull *ADCY10* variant I mRNA, Figure 1C and Figure 3] [31]. The bull variant II coding the ADCY10_tf_ lacks exon 11 due to the alternative splicing instead of exon 12 (Figure 1C and Figure 3). These results show that the site of the alternative splicing for the derivation of ADCY10_tf_ differs between bulls and rodents. Moreover, the bull *ADCY10* genome sequence in the Ensemble database (gene ID: ENSBTAG00000001052, http://asia.ensembl.org/index.html) indicate that the 3′ terminus of the intron connecting to exon 10 is composed of the nucleotides “^−10^CCGGGAGCCAG^−1^”. It is likely that the splicing error occurring near exon 10 is due to an incorrect selection of the intronic sequences (AG) located at the –6 and –5 positions from the initial part of exon 10 (Figure 3).

With the characteristic analyses of *ADCY10* variant mRNAs collected from bull testes, we found that the alternative splicing for exon 11 is important in the derivation of transcripts coding the bull ADCY10_tf_ protein, and that the serious splicing errors occur at the 3′ splice site of the intron connecting to the initial part of exon 10.

**Figure 4:**
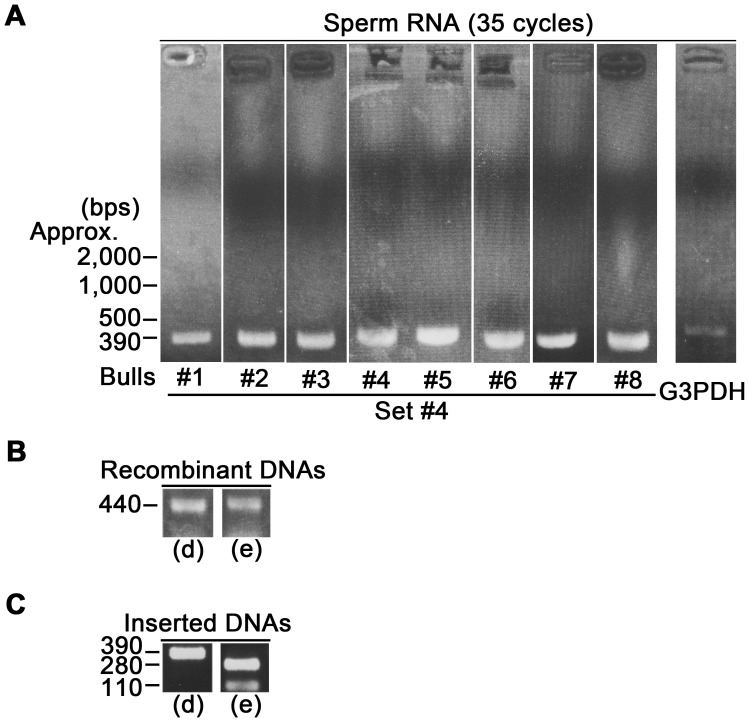
Detection of vestiges of splicing errors using residuals of testicular mRNAs in bull spermatozoa. A: The products amplified by RT-PCR with total RNA from spermatozoa and primer set #4 were visualized with a transilluminator. **B:** Twenty-six recombinants into which the products amplified by RT-PCR with primer set #4 and RNA from frozen-thawed ejaculated spermatozoa were inserted were subjected to colony PCR using a forward primer for a *pSPT19* plasmid and a reverse primer of a primer set #4, in order to check the successful ligation. The molecular size of colony PCR products is larger by approximately 50 bases than that of PCR products (see panel A), because the colony PCR products contain the nucleotides coded by plasmids. The nucleotide sequences of the inserted parts of obtained recombinants were revealed by sequential analyses. Consequently, the recombinants coded variant II [the representative of 16 recombinants: (d)] or variant III [the representative of 10 recombinants: (e)]. **C:** Inserted DNAs of the 26 recombinants were treated with a restriction enzyme (*Cac8I*). Consequently, 16 recombinants coding variant II without the cleavage site for *Cac8I* were detected as the single band [representative: (d)]. Ten recombinants coding variant III with the cleavage site for *Cac8I* were detected as two bands [representative: (e)].

In the next experiment, we attempted to detect the vestige of the above-mentioned splicing error in the residual mRNAs from spermatozoa, to find an alternative sample source for testicular mRNAs. According to previous report [33], it is likely that RNAs of ejaculated spermatozoa without the cytoplasmic droplet largely include products of testicular RNA fragmentation. In fact, the full-length 18S and 28S ribosomal RNAs are highly fragmented and undetectable in mature spermatozoa [33], [34]. However, Figure 4A shows that only an approximately 390-bp DNA fragment could specifically be amplified by RT-PCR with primer set #4 and sperm RNA, and that almost no large difference in the electrophoresis band of the PCR product was observed between bulls. From these results, we inferred that all of sperm samples used in this study preserved enough RNA fragments to detect the above-mentioned splicing error in bull *ADCY10* mRNAs by RT-PCR. In the preliminary experiments to confirm no contamination with the genomic DNA in the sperm RNA, we amplified the fragments coding exons 10 and 11 of bull *ADCY10* by RT-PCR, using sperm RNA and five kinds of primer sets (#13–#17) (Figure S2A and Table S1). Since the molecular size of intron part between exons 10 and 11 was approximately 2.2 kb (NW_001494712), possible PCR products derived from the genomic DNA might be supposed to appear at the +2.2 kb upper positions in the electropherogram. As shown in the Figure S2C, however, no band was detected at the +2.2 kb upper positions. The supporting results were also shown in Figure 4A. In order to identify the variant types of the amplified products, we randomly selected 26 recombinants into which the amplified products were inserted [Figure 4B, representatives (d) and (e)], and we subsequently analyzed the nucleotide sequences of the inserted parts of the recombinants.

All of the analyzed recombinants coded variant II [the representative of 16 recombinants: (d)] or III [the representative of 10 recombinants: (e)] (Figure 4B). The reason for no variant I in the sperm mRNA was explained by the preliminary experiment in which we amplified the fragments coding exons 10 and 11 of bull *ADCY10* by RT-PCR using primer sets #13–17 and testicular or sperm RNA. As shown in Figure S2 panels B and C, the PCR products amplified with primer sets #13–#16 were detected in testes and spermatozoa. The PCR product amplified with primer set #17 was detected in only testes. These results indicate that most of the transcripts coding variant I in spermatozoa are fragmented at the second half of exon 11. However, mRNA fragments of variants II and III coding exons 9, 10, 12 and 13 were preserved in the spermatozoa by evading the fragmentation at the exon 11 and could be amplified as enough cDNA fragments by RT-PCR using the primer set #4 (Figure 4A). This allows us to detect vestiges of the splicing error near exon 10 of *ADCY10* mRNA in the residuals of testicular mRNAs collected from the ejaculated spermatozoa.

The restriction enzyme *Cac8I* was able to selectively digest variant III containing the nucleotide sequence [G+CCAG+C; the 3′ terminus of exon 9 (1 base)+intronic nucleotides (4 bases) retaining due to the splicing error+the 5′ terminus of exon 10 (1 base; see Figure 3)] but not variant II (Figure 4C). This nucleotide sequence corresponds to the specific cleavage site for *Cac8I* (GCN/NGC, http://www.neb.com/nebecomm/products/productr0579.asp). These results indicate that the digestion treatment with *Cac8I* can probe the splicing errors that cause the derivation of variant III.

**Figure 5:**
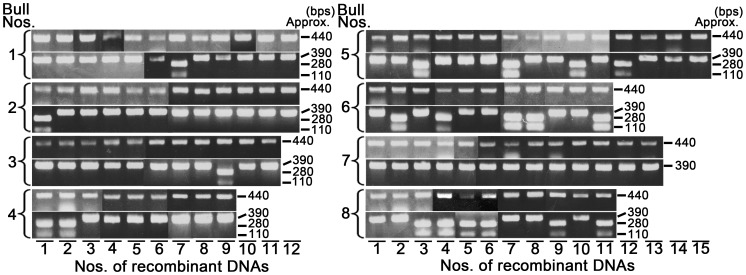
Construction of recombinants using the sperm RNA from eight bulls and *Cac8I* digestion treatment. The recombinants obtained by cloning methods using frozen-thawed ejaculated spermatozoa (from bulls #1–#6), freshly epididymal spermatozoa (from bull #7) and freshly ejaculated spermatozoa (from bull #8) were used for colony PCR (upper panels for each bull), and then the inserted parts of recombinants were digested by *Cac8I* (lower panels).

**Figure 6:**
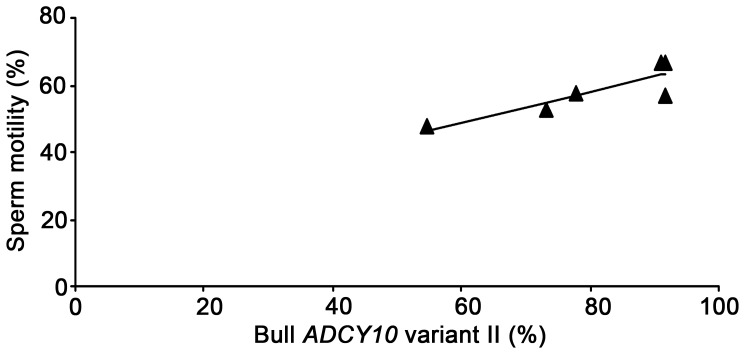
Examination of the relationship between the splicing accuracy in *ADCY10* transcription process and sperm motility. The percentages of variant II mRNAs in frozen-thawed ejaculated spermatozoa positively correlated with those of motile spermatozoa immediately after thawing (n = 6, R = 0.87, p = 0.025).

Thus, in the final experiment to estimate the individual variation of the frequency of splicing errors, we applied this digestion treatment to the sperm samples collected from eight bulls. As shown in Table 1 and Figure 5, the percentages of the recombinants with the *Cac8I* cleavage site were varied markedly, between 0% and 54.5%. These results suggest the existence of large individual differences in the accuracy of mRNA splicing in testes. Moreover, it is very interesting that the percentages of the recombinants without the specific cleavage site for *Cac8I* (namely without the intronic nucleotides due to the splicing errors) were positively correlated with those of sperm motility (R = 0.87, p = 0.025, Figure 6) or those of the progressively motile spermatozoa (R = 0.88, p = 0.020, data not shown). The exact relationship between these parameters remains to be examined.

**Table 1:** Typing of the residual fragments of *ADCY10* variant mRNAs in bull spermatozoa and the percentages of sperm motility.

Bulls	Total No. of examined recombinants	No. of recombinants (%)		Average of sperm motility before washing (%)¶	
(Date of the sperm collection)		Without *Cac8I* cleavage site*	With *Cac8I* cleavage site**	Motility	Progressive motility
				(No. of observations)	
#1†,‡	12	11 (91.7)	1 (8.3)	57.0	31.0
(12. May. 2011)				(n = 6)	
#2	12	11 (91.7)	1 (8.3)	66.7	30.0
(2. Jul. 2007)				(n = 3)	
#3‡	11	10 (90.9)	1 (9.1)	66.7	23.3
(27. Oct. 2009)				(n = 3)	
#4	9	7 (77.8)	2 (22.2)	57.5	27.5
(11. May. 2006)				(n = 4)	
#5‡	15	11 (73.3)	4 (26.7)	52.5	20.0
(2. Dec. 2003)				(n = 4)	
#6†	11	6 (54.5)	5 (45.5)	47.5	12.5
(18. May. 2006)				(n = 4)	
#7	13	13 (100.0)	0 (0.0)	60.0	0.0
(24. Mar. 2011)				(n = 1)	
#8	11	5 (45.5)	6 (54.5)	30.0	0.0
(8. Jun. 2011)				(n = 1)	

Ejaculated spermatozoa (from bulls #1–#6) were used for the analyses after the frozen storage for the artificial insemination. Epididymal spermatozoa (from bull #7) after washing were stored in liquid nitrogen until they were used for the analyses. In addition, ejaculated spermatozoa (from bull #8) were also analyzed without the frozen storage.

† The motility of frozen-thawed ejaculated spermatozoa (from bulls #1 and #6) before washing was recorded (see Movies S2 and S3).

‡ The motility of spermatozoa (from bulls #1, #3 and #5) immediately after thawing was also evaluated by Sperm Motility Analysis System (see Table S2).

* Recombinants without the cleavage site for *Cac8I* indicate that these recombinants code *ADCY10* variant II mRNAs.

** Recombinants with the cleavage site for *Cac8I* indicate that these recombinants code *ADCY10* variant III mRNAs.

¶ In bulls #1–#6, the subjective observations of motility were done for frozen spermatozoa collected from each of 3–6 straws. In bulls #7 and #8, the subjective observations of motility were done for unfrozen spermatozoa.

In order to confirm exactness in the conventional (but general) motility assay for livestock spermatozoa, we analyzed the motility of frozen-thawed ejaculated spermatozoa (from bulls #1, #3 and #5) using SMAS. Individual differences in motility score shown by SMAS assay (Table S2) were detectable by the conventional assay (Table 1).

Moreover, bull ADCY10 variant ortholog proteins existed in apical edges of acrosomes, post-acrosomal regions, connecting pieces and principal pieces of flagella in spermatozoa (Type I) (Figure S3, Panels a and d), or in equatorial segments, connecting pieces and principal pieces of flagella in spermatozoa (Type II) (Figure S3, Panels b and e). The rates of spermatozoa classified into the Types I and II were 87.5% and 12.5%, respectively. These results indicate that the bull ADCY10 ortholog proteins are mainly distributed in ejaculated spermatozoa as shown in the Type I, and that all of the ejaculated bull spermatozoa possess ADCY10 ortholog proteins in the principal pieces. In the principal piece, the increased cAMP promotes protein serine/threonine phosphorylation of the dynein-ATPase and other axonemal proteins via activation of protein kinase A and then modulates flagellar beating [35]–[37]. Previous reports demonstrated that seminal plasma bicarbonate initiates and stimulates progressive motility of ejaculated spermatozoa by the activation of flagellar adenylyl cyclase and consequently increase of the intracellular cAMP [38], [39]. These findings are consistent with our suggestion that ADCY10 of principal pieces may have the function as the synthesizer of the cAMP to modulate the motility in ejaculated bull spermatozoa.

In conclusion, to our knowledge, this is the first report showing the utility of residual sperm mRNA to detect the vestiges of the occurrence of splicing errors of mRNA in mammalian testes. This method may potentially be applied to the estimation of accuracy of the mRNA splicing in the testes for various species including livestock and human.

## Supporting Information

Figure S1 **Amino acid sequences of the protein coded by the bull** ***ADCY10*** **variant I.** The asterisks are identical amino acids between the protein coded by the bull *ADCY10* variant I and human ADCY10 protein (accession# NP_060887) or the rat ADCY10 protein (NP_067716). Hyphens indicate the lacking parts of the corresponding amino acids between these sequences. Underlined sequences indicate the cyclase domains of the bull ADCY10 predicted by the database analyses with CD-search. The sequential homology rates of the protein coded by the bull *ADCY10* variant I were 80% and 81% against the human ADCY10 protein and the rat ADCY10 protein, respectively.
(DOC)

Figure S2 **Examination of the amplification of nucleotide sequences coding exons 10–11 of the bull** ***ADCY10*** **.** A: Five primer sets (#13–#17) were designed to amplify exons 10–11 of the bull *ADCY10* variant I. The numbers surrounded by parentheses are the expected molecular sizes of PCR products. The sequential numbers indicate the nucleotide numbers in exon 10 or exon 11. **B:** The RT-PCR products amplified with testicular RNA and primer sets #13–#17 were separated in 3% agarose gels containing 0.01% ethidium bromide (EtBr). *Glycerol-3-phosphate dehydrogenase* (*G3PDH*) was used as the control. The cycle number was 30. (The panel is representative of three replicates). **C:** The RT-PCR products amplified with RNA from freshly epididymal spermatozoa (from bull #7) and primer sets #13–#17 were visualized with a transilluminator. The cycle number was 40. (The panel is representative of three replicates).
(TIF)

Figure S3 **Immunodetection of bull ADCY10 variant ortholog proteins in freshly ejaculated spermatozoa by indirect immunofluorescence (representative of four replicates).** Ejaculated spermatozoa from two bulls were treated with paraformaldehyde, Triton-X and subsequently PBS containing bovine serum albumin. These samples were subjected to the mouse antiserum (1∶30) and then the FITC-conjugated rabbit anti-mouse immunoglobulins antibody (1∶50) (Panels a – b and d – e). In the control experiment, NMS was used instead of the primary antibody (Panels c and f). A bar scale on the panel shows 20 µm.
(TIF)

Table S1 **Primer sets designed to amplify bull** ***ADCY10*** **variant mRNAs.**
(DOC)

Table S2 **Analyses of sperm motility using Sperm Motility Analysis System (SMAS).** The motility of frozen-thawed ejaculated spermatozoa (from bulls #1, #3 and #5) that were used for the collection of residual fragments of testicular *ADCY10* variant mRNAs (see Table 1) was evaluated by SMAS. Specifically, the sperm suspension was adjusted the concentration to 1×10^7^ cell/mL with PBS-PVA. Five-µL aliquot of this suspension was put on the 20-µm chamber slide and subsequently sperm motility was recorded on a heated stage at 38.5°C under a phase contrast microscope. Approximately 5 fields per preparation were randomly selected, and then the sperm motility was evaluated by *in silico* analyses using a developing parameter for livestock. The analyses were replicated twice for each bull.
(DOC)

Movie S1 **Observation of spermatozoa after Percoll purification.** The frozen-thawed ejaculated spermatozoa (from bull #2) were washed once in a two-step gradient of 2 mL of 60% and 5 mL of 30% isotonic Percoll that was prepared with PBS, and subsequently washed once in PBS-PVA by centrifugation. After centrifugation, the sperm pellet was suspended with PBS-PVA and observed on a heated stage at 38.5°C under a bright-field microscope.
(WMV)

Movie S2 **Observation of the motility of frozen-stored ejaculated spermatozoa (from bull #1) immediately after thawing.**
(WMV)

Movie S3 **Observation of the motility of frozen-stored ejaculated spermatozoa (from bull #6) immediately after thawing.**
(WMV)
