# Supplementary material for: Novel Approach for the Detection of the Vestiges of Testicular mRNA Splicing Errors in Mature Spermatozoa of Japanese Black Bulls
Source: PLoS One. 2013 Feb 26;8(2):e57296. doi: 10.1371/journal.pone.0057296 (PMC3582612; doi:10.1371/journal.pone.0057296)
Supplement: Figure S1 — Amino acid sequences of the protein coded by the bull ADCY10 variant I. The asterisks are identical amino acids between the protein coded by the bull ADCY10 variant I and human ADCY10 protein (accession# NP_060887) or the rat ADCY10 protein (NP_067716). Hyphens indicate the lacking parts of the corresponding amino acids between these sequences. Underlined sequences indicate the cyclase domains of the bull ADCY10 predicted by the database analyses with CD-search. The sequential homology rates of the protein coded by the bull ADCY10 variant I were 80% and 81% against the human ADCY10 protein and the rat ADCY10 protein, respectively. (DOC) [file pone.0057296.s001.doc]

|  | |  | | |
| --- | --- | --- | --- | --- |
| Bull | 1 | MNTRRELESQDRAIVKIAAHLPDLIVYGDFSPERPSVDNFDGVLMFVDISGFTAMTEKFSTAMYMDRGAEQLVEILNHYISAIVEKVLIFGGDILKFAGDALLALWKVERKQLKNIITVVIKCSLEIHGLFETQESEEGL | | |
| Human | 1 | ***PK*-*F**WP**R************H******FM*Y*********************S****************YH***************************R****************************W**** | | |
| Rat | 1 | *SA**Q-*L****************************KC**************************************Y******************************************************AK*V**** | | |
|  | |  |  |  |
| Bull | 141 | DIRVKIGLAAGHISMLVFGDETRNYFLVIGQAVDDVRLAQNMAQVNDVILSPNCWQLCDRSMIEIERIPDQRAVKVNFLKPPPSFNFDEFFTKCMAFMDYYPSGDHKNLLRLACMLESDPELELSLQKYVMESILKQIDD | | |
| Human | 140 | **********************HSH*******************M*********************SV***************N**********TT**H*****E*********T*KP*****M***************N | | |
| Rat | 140 | *************T******************************M*******************************S******T*******A****************F***********************I******* | | |
|  | |  |  |  |
| Bull | 281 | KQLRGYLSELRPVTVMFVNLMFKDQDKAEVIGSAIQDACVHINSVLRVFQGQINKVFMFDKGCSFLCVFGFPGEKAPDEITHALESAVDIFDFCSQVHKIRTVSIGVTSGIVFCGIVGHSVRHEYTVIGQKVNIAARMMM | | |
| Human | 280 | ***Q**********IV******E******E**P*****YM**T***KI***************************V***L*****C*M************Q******A***********T*************L****** | | |
| Rat | 280 | **************IV*******E************A*****T***K**R*********************************************************A***********T******************** | | |
|  | |  |  |  |
| Bull | 421 | YYPGIVTCDSVTYNGSNLPAYFFKELPKKVMKGVADSGPVYQCLGLNEKVMFGMAYLICNRNERYPLLGREEEIKYFMCTMKKFLMSNCSQVLMYEGISGYGKSQILVEIEYLAHG-ENHRTIAIVLTKISFNQNFYTIQ | | |
| Human | 420 | ***************************************L**YW*RT********C*****K*D******NK**N***Y******I**S********LP********MK*****Q*-K***I***S*N****H*T***** | | |
| Rat | 420 | *************D**********************P************************Y*G******VR**D***S***D***T***R******LP******V*M******SQH****AV**A******H******* | | |
|  | |  | | |
| Bull | 560 | ILMANVLGLDTCKHYKERQTNLQNKVKSLLDEKFHCLLNDIFHVQFPISREVSKMSTMRKQKQLEALFMKILEQTVKEERIIFIIDEGQFIDSASWTFMEKLIRSVPIFIIMSLSPFVNTPCAAASAIMKNRNTTYVTLG | | |
| Human | 559 | MF********************R***MT******Y****************I*R***LK******I******KLI************A**V**T**R*******TL********C****I*****R*VI*******IVI* | | |
| Rat | 560 | ************************R**T***D*Y*************V***M*R**KI******************R**********A**V*V***A*I******M****V***C**PE******N**********I*** | | |
|  | |  |  |  |
| Bull | 700 | PVQPKDILNKVCLDLSVKGIPKELDTYLAEGSCGIPFYCEELLKNLDHHRVLVFQAMESEEKTNVTWNNLFKNFAKPTEDLKTFTFSLEEENEEVCNLASGVRLKNLSPPASLKEISLVQLDSMSLSHQMLVRCAAIIGL | | |
| Human | 699 | A***N**S**I****N*SC*S****S**G*****************E**E*****QT*******R*******YSI*L**K*NMV*LHSDK*S****H*T***********T*******I*****R*************** | | |
| Rat | 700 | TM**QE*RD***V****SS**R***S**V*******Y*************I*I**QA*A*************YSV*****MYLY*-*IAAGQK*A*Y*T***************************************** | | |
|  | |  |  |  |
| Bull | 840 | TFTTELLFEILPCWDMKMMINALATLVESNIFDCFQNGKELRMALEKNAASFEVNYRSLSLKPLIEGMDHGEEEQLRELESEVIQCHIIRFCSPVMQKTAYELWLKDQKKAMHLKCARFLEENAHRCEPCRSGDFVPFHH | | |
| Human | 839 | **************N*****KT**********Y**R*****QK**KQ*DP****H*******-PS***************N***E**R****N*M*************R*************D****DH**GR**I*Y** | | |
| Rat | 839 | **************N*****K*********V****RSS*D*QL**KQ*VTT***H*******-SK**LAYS*******M*G***E*R*L***R*I***************VL**********S****NH**NR**I*Y** | | |
|  | |  |  |  |
| Bull | 980 | FAVDIRLNTLDLDTIRKMAKSHGFRTEEEITFSRIETSKKSEIFSENLSSEEIREKILSFFDNIITKMKMSEDNVIPLESCQCEEILEIVIMPLAHHFLALEENNKALYYFLELVSAYLVLGDNYMAYLYLNEGERLLKI | | |
| Human | 978 | *T*N****A**M*A*K***M****K***KLIL*NS*IPET*AF*P**R*P********N***HVL****T*DEDI****************L*********G**D********IA****IFC******M*****QK***T | | |
| Rat | 978 | *IA********M**VK**V*****K**D*VI**KS*IPR*-FK*P**I*IT*T*****H****V*I**RT*Q*D*******H***L*Q***L***Q**V***************A****I*****N**M**G*******S | | |
|  | |  |  |  |
| Bull | 1120 | LKKDKSWSKTFEWATFFTLKGQVCFNMGQMVLAKKMLRKALKFLNRIFPYNLISLFLQTRVEKNRHFHYVNQNQQAQESSPPGKKRLAQLYQQTTCFSLLWQIYSLNFFFHYKYYCHLAALMQVNTALETQDDFQIIKAY | | |
| Human | 1118 | ********Q***S***YS***E*******I************L**************HIH***********--R*****P***********R**V*L****R***YSYL**C***A***VM**M*******NC******* | | |
| Rat | 1117 | *TNED***Q***Y***YS***EI*******************L***M**C**L**TF*MHI****LS*FM*--*HT**G*L****-****FL*SS******K*************GR***I**M**S****NN******F | | |
|  | |  |  |  |
| Bull | 1260 | LDYSLYQHLAGYQDVWFKYEVMAMKQIFNLPLKREGVEIMTYVADRLGYIKLLMGQLDLAIDLGSRAHKMWSLLRNPNKHYLVLCWLCKSLFLKNRYKQLIQVLGWLWDLSVAEDHIFSRAFFYFICLDIMLYSGFVYRP | | |
| Human | 1256 | ******H*****KG**********EH*******G**I**VA***ET*VFN**I**H*****E*****LQ**A**Q***R**QS**R*SRC*L*NS**P*******R**E***TQE****K*****V****L********T | | |
| Rat | 1254 | **F***R*****EG******ILV*E*LL*****G*AF***A*A**A**H**F*T*H*****E*****************YHM***R*S*P****S***H*V***********T*E****K*****V**********I**T | | |
|  | |  |  |  |
| Bull | 1400 | FEECLQFIHQNEDNRLLKFQSGMLLGLYSCIAIWYARLQEWNSFRVFSNRAKNLVSRRTPTALYCISISRYMEGQVLYLQKQIEEQSENAQDAGVDLLKNLENLVAQNTTGPVFYPRLYHLMAYVCILMGEGQNCDLFLN | | |
| Human | 1396 | *****E****Y*N**I***H**L******SV**********DN*YK********LP***M*LT*YDG**********H*****K*******AS*EE******************C***************D**K*G**** | | |
| Rat | 1394 | *****E***H*****I******L*********V********DN*YK******T**T*****V**YEG**********H********A*****S**E***A**T***************************D*HS**F*** | | |
|  | |  |  |  |
| Bull | 1540 | TALQFCETQGNVLEKCWLNMSKEWWYSNSELTGDQWLQTLLNLPSWEKVVSGKVNIQDIQKNKFLMRVNILDNPF | | |
| Human | 1536 | ***RLS*****I********N**S***T***KE******I*S******I*A*R*****L********A*TV**H* | | |
| Rat | 1534 | ***ELS*****L******S********AP**********V*S****D*I***N*TL**V**************** | | |

**Figure S1.**
