# Supplementary figures and images for: Novel Approach for the Detection of the Vestiges of Testicular mRNA Splicing Errors in Mature Spermatozoa of Japanese Black Bulls
Source: PLoS One. 2013 Feb 26;8(2):e57296. doi: 10.1371/journal.pone.0057296 (PMC3582612; doi:10.1371/journal.pone.0057296)

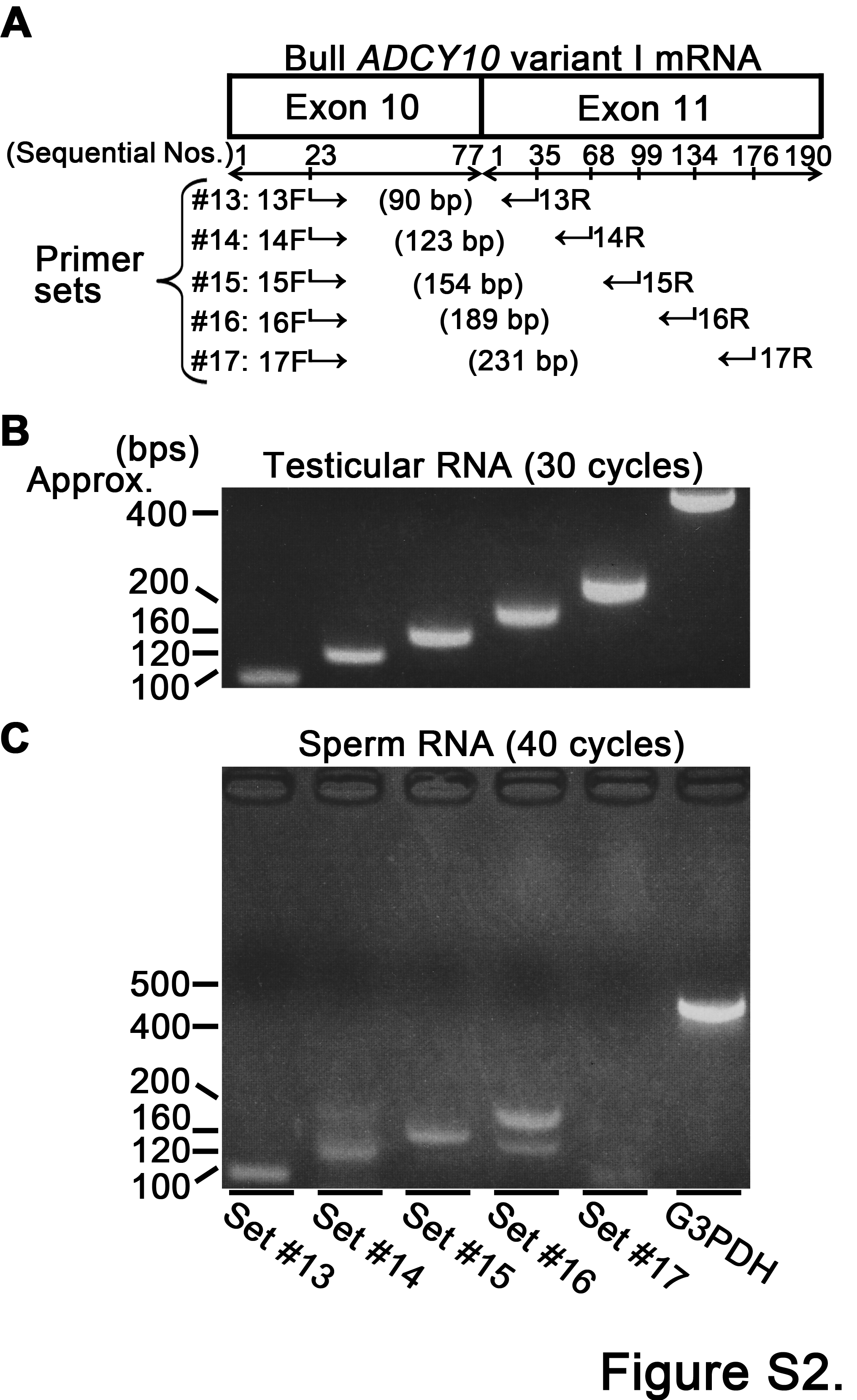

Supplement: Figure S2 — Examination of the amplification of nucleotide sequences coding exons 10–11 of the bull ADCY10 . A: Five primer sets (#13–#17) were designed to amplify exons 10–11 of the bull ADCY10 variant I. The numbers surrounded by parentheses are the expected molecular sizes of PCR products. The sequential numbers indicate the nucleotide numbers in exon 10 or exon 11. B: The RT-PCR products amplified with testicular RNA and primer sets #13–#17 were separated in 3% agarose gels containing 0.01% ethidium bromide (EtBr). Glycerol-3-phosphate dehydrogenase (G3PDH) was used as the control. The cycle number was 30. (The panel is representative of three replicates). C: The RT-PCR products amplified with RNA from freshly epididymal spermatozoa (from bull #7) and primer sets #13–#17 were visualized with a transilluminator. The cycle number was 40. (The panel is representative of three replicates). (TIF) [file pone.0057296.s002.tif]

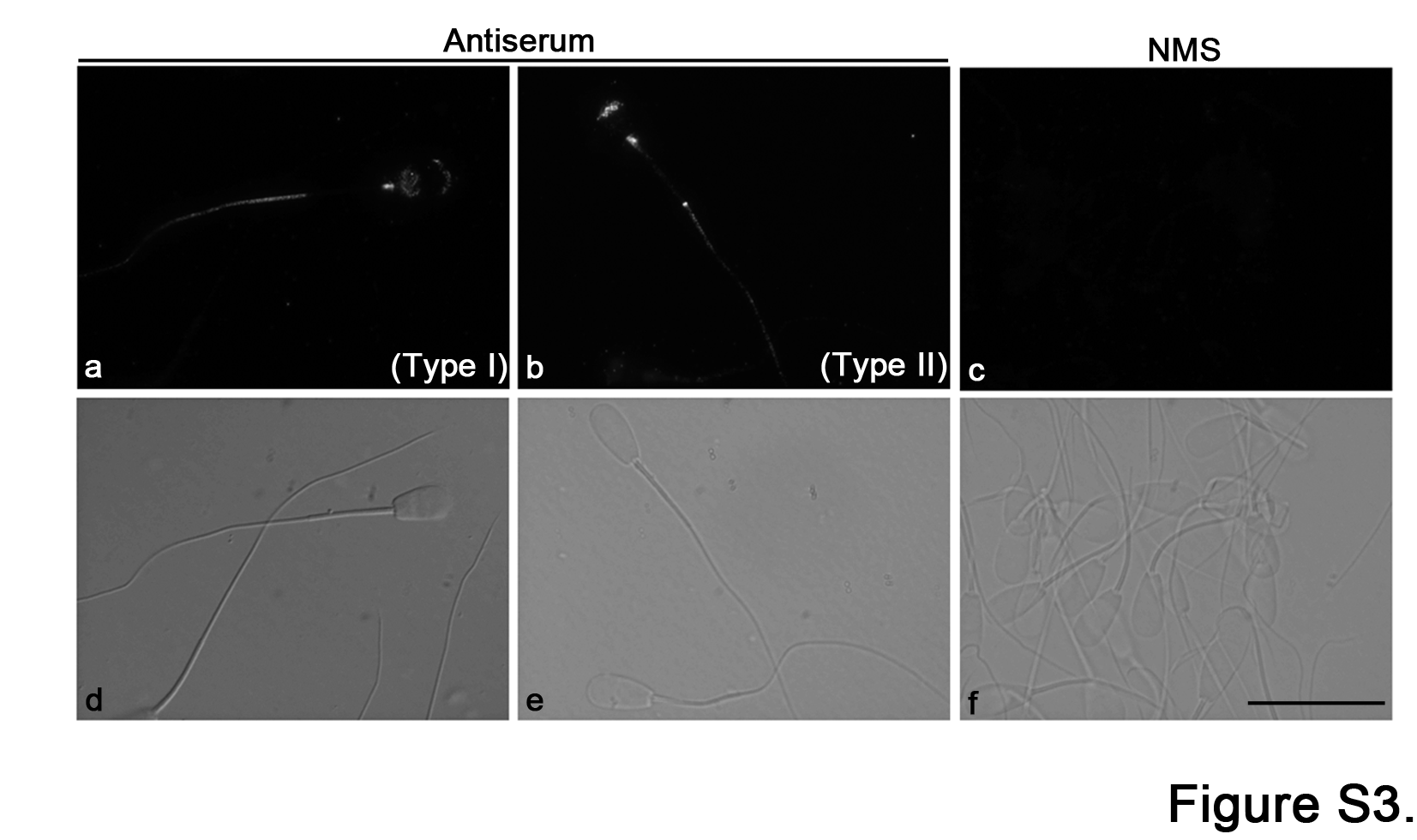

Supplement: Figure S3 — Immunodetection of bull ADCY10 variant ortholog proteins in freshly ejaculated spermatozoa by indirect immunofluorescence (representative of four replicates). Ejaculated spermatozoa from two bulls were treated with paraformaldehyde, Triton-X and subsequently PBS containing bovine serum albumin. These samples were subjected to the mouse antiserum (1∶30) and then the FITC-conjugated rabbit anti-mouse immunoglobulins antibody (1∶50) (Panels a – b and d – e). In the control experiment, NMS was used instead of the primary antibody (Panels c and f). A bar scale on the panel shows 20 µm. (TIF) [file pone.0057296.s003.tif]
