# Supplementary material for: Novel Approach for the Detection of the Vestiges of Testicular mRNA Splicing Errors in Mature Spermatozoa of Japanese Black Bulls
Source: PLoS One. 2013 Feb 26;8(2):e57296. doi: 10.1371/journal.pone.0057296 (PMC3582612; doi:10.1371/journal.pone.0057296)
Supplement: Table S1 — Primer sets designed to amplify bull ADCY10 variant mRNAs. (DOC) [file pone.0057296.s004.doc]

| Primers | Forward primers | | Reverse primers | | PCR products |
| --- | --- | --- | --- | --- | --- |
|  | Nucleotide sequences | Sequential Nos. | Nucleotide sequences | Sequential Nos. | Expected molecular sizes |
| Set #1 | TGGCACTTCTGCTGTCTTCTG | 121-141^†^ | CTTGGAAGACCCTCAGGACA | 1301-1282^†^ | 1181 |
| Set #2 | TCTATCCCCAAACTGCTGGC | 877-896^†^ | TTGTGGACCTGAGAGCAGAAG | 1445-1425^†^ | 569 |
| Set #3 | GGCTCAGCTATTCAGGATGC | 1244-1263^†^ | AAGTAGGCTGGTAGGTTGCTG | 1634-1614^†^ | 391 |
| Set #4 | GGAATTCTGTCTTTGGTTTCCTGGGG^††^ | 1351-1369^†^ | GCGAAGCTTGTGGGCCAGGTACTCAATTT^‡‡^ | 1914-1895^†^ | 580 |
| Set #5 | CTCTGTCACCTACAATGGCAG | 1596-1616^†^ | GCTGAATCGATAAACTGGCCC | 2267-2247^†^ | 672 |
| Set #6 | GCAGTTCCCTATTTCACGGG | 2118-2137^†^ | GATTCCACAAGGGTTGCC | 2912-2895^†^ | 795 |
| Set #7 | CGCTAGTGGTGTCAGATTGAA | 2700-2720^†^ | GCGAAGTGATGAAAGGGGAC | 3251-3232^†^ | 552 |
| Set #8 | GCTTTCTGGAAGAAAACGCC | 3179-3198^†^ | GCTCTCTTGGGCCTGTTGA | 3903-3885^†^ | 725 |
| Set #9 | CCAGATGGTGCTTGCCAAA | 3750-3768^†^ | CTCCAGCGTCTTGAGCATTC | 4789-4770^†^ | 1040 |
| Set #10 | CAGGAATGGAACAGCTTTCG | 4621-4640^†^ | GTTAGAAAGGATTGTCCAGAA | 5153-5133^†^ | 533 |
| Set #11^¶^ | CGAATTCCAGAATATTTGCCTTGAACA^††^ | 290-309^†^ | GTCAAGCTTGCTGAGCCTATGACTTCTGCT^‡‡^ | 1251-1231^†^ | 978 |
| Set #12^*^ | CGAATTCCTGAAGAAGGCCTGGATATC^††^ | 435-454^‡^ | GGGAAGCTTTGGGCGAAGCTCAGACAA^‡‡^ | 904-887^‡^ | 486 |
| Set #13^**^ | AGCGGGATTGTCTTCTGTGG | 1193-1212^‡^ | CATTCTGGCAGCTATGTTGAC | 1282-1262^‡^ | 90 |
| Set #14^**^ | AGCGGGATTGTCTTCTGTGG | 1193-1212^‡^ | GTCACAGGTTACGATTCCTGG | 1315-1295^‡^ | 123 |
| Set #15^**^ | AGCGGGATTGTCTTCTGTGG | 1193-1212^‡^ | CTGGTAGGTTGCTGCCATTG | 1346-1327^‡^ | 154 |
| Set #16^**^ | AGCGGGATTGTCTTCTGTGG | 1193-1212^‡^ | CATGACTTTCTTTGGAAGCTC | 1381-1361^‡^ | 189 |
| Set #17^**^ | AGCGGGATTGTCTTCTGTGG | 1193-1212^‡^ | GCCCAAACACTGATACACTGG | 1423-1403^‡^ | 231 |

Table S1. Primer sets designed to amplify bull *ADCY10* variant mRNAs.

^†^The sequence numbers in primer sets #1 – #11 indicate the nucleotide numbers in the putative bull *ADCY10* cDNA sequence which was estimated by discontiguous megaBLAST analyses against the genome and HTGS databases of *Bos taurus* and human *ADCY10* cDNA sequence as the query.

^‡^The sequence numbers in primer sets #12 – #17 indicate the nucleotide numbers in the bull *ADCY10* variant I (accession number: AB735412) which was revealed by the nucleotide sequencing in this study.

^††^The nucleotide sequences include the restriction enzyme site for *EcoRI* (GAATTC).

^‡‡^The nucleotide sequences include the restriction enzyme site for *HindIII* (AAGCTT).

^¶^The primer set #11 was used for the production of cRNA probes for Northern blotting.

^*^The primer set #12 was designed to amplify the nucleotide sequence coding the first cyclase domain of bull ADCY10 (AB735412, the corresponding region: amino acid #136 – #292) and used for the antibody production.

^**^The primer sets #13 – #17 were used for the amplification of nucleotide sequences coding exons 10 and 11 in bull *ADCY10* variant I.
