# Supplementary material for: Novel Approach for the Detection of the Vestiges of Testicular mRNA Splicing Errors in Mature Spermatozoa of Japanese Black Bulls
Source: PLoS One. 2013 Feb 26;8(2):e57296. doi: 10.1371/journal.pone.0057296 (PMC3582612; doi:10.1371/journal.pone.0057296)
Supplement: Table S2 — Analyses of sperm motility using Sperm Motility Analysis System (SMAS). The motility of frozen-thawed ejaculated spermatozoa (from bulls #1, #3 and #5) that were used for the collection of residual fragments of testicular ADCY10 variant mRNAs (see Table 1) was evaluated by SMAS. Specifically, the sperm suspension was adjusted the concentration to 1×10^7^ cell/mL with PBS-PVA. Five-µL aliquot of this suspension was put on the 20-µm chamber slide and subsequently sperm motility was recorded on a heated stage at 38.5°C under a phase contrast microscope. Approximately 5 fields per preparation were randomly selected, and then the sperm motility was evaluated by in silico analyses using a developing parameter for livestock. The analyses were replicated twice for each bull. (DOC) [file pone.0057296.s005.doc]

| Bulls | Total No. of cells (No. of observations) | | Percentages of the motility of spermatozoa immediately after thawing (average ± standard deviation) |
| --- | --- | --- | --- |
|  | Motility | Immobility |  |
| #1 | 140 (n=11) | | 70.34 ± 8.37 |
|  | 99 | 41 |  |
| #3 | 138 (n=11) | | 69.01 ± 14.59 |
|  | 98 | 40 |  |
| #5 | 90 (n=10) | | 57.38 ± 19.05 |
|  | 52 | 38 |  |

Table S2. Analyses of sperm motility using Sperm Motility Analysis System (SMAS).

The motility of frozen-thawed ejaculated spermatozoa (from bulls #1, #3 and #5) that were used for the collection of residual fragments of testicular *ADCY10*

variant mRNAs (see Table 1) was evaluated by SMAS. Specifically, the sperm suspension was adjusted the concentration to 1 × 10^7^ cell/mL with PBS-PVA.

Five-μL aliquot of this suspension was put on the 20-μm chamber slide and subsequently sperm motility was recorded on a heated stage at 38.5°C under a phase contrast microscope. Approximately 5 fields per preparation were randomly selected, and then the sperm motility was evaluated by *in silico* analyses using a developing parameter for livestock. The analyses were replicated twice for each bull.
